# Opportunities and challenges of Integral Projection Models for modelling host–parasite dynamics

**DOI:** 10.1111/1365-2656.12456

**Published:** 2015-12-01

**Authors:** C. Jessica E. Metcalf, Andrea L. Graham, Micaela Martinez‐Bakker, Dylan Z. Childs

**Affiliations:** ^1^ Department of Ecology and Evolutionary BiologyPrinceton UniversityPrincetonNJUSA; ^2^ Office of Population ResearchThe Woodrow Wilson SchoolPrinceton UniversityPrincetonNJUSA; ^3^ Department of Ecology and Evolutionary BiologyUniversity of MichiganAnn ArborMIUSA; ^4^ Department of Animal and Plant SciencesSheffield UniversitySheffieldUK

**Keywords:** demography, dynamics, infectious disease, Integral Projection Model, measles, murine malaria, parasite

## Abstract

1. Epidemiological dynamics are shaped by and may in turn shape host demography. These feedbacks can result in hard to predict patterns of disease incidence. Mathematical models that integrate infection and demography are consequently a key tool for informing expectations for disease burden and identifying effective measures for control.
2. A major challenge is capturing the details of infection within individuals and quantifying their downstream impacts to understand population‐scale outcomes. For example, parasite loads and antibody titres may vary over the course of an infection and contribute to differences in transmission at the scale of the population. To date, to capture these subtleties, models have mostly relied on complex mechanistic frameworks, discrete categorization and/or agent‐based approaches.
3. Integral Projection Models (IPMs) allow variance in individual trajectories of quantitative traits and their population‐level outcomes to be captured in ways that directly reflect statistical models of trait–fate relationships. Given increasing data availability, and advances in modelling, there is considerable potential for extending this framework to traits of relevance for infectious disease dynamics.
4. Here, we provide an overview of host and parasite natural history contexts where IPMs could strengthen inference of population dynamics, with examples of host species ranging from mice to sheep to humans, and parasites ranging from viruses to worms. We discuss models of both parasite and host traits, provide two case studies and conclude by reviewing potential for both ecological and evolutionary research.

## Introduction

Over the course of an infection, as the parasite replicates and evades or overcomes the host's defences, parasite density, size or abundance and associated immune responses fluctuate, often following complex trajectories (Metcalf *et al*. 2011). These fluctuations shape host and parasite population‐level outcomes via their effects on rates of host recovery, pathology, between‐host transmission and waning of host immunity (Gilchrist, Coombs & Perelson 2004; Graham *et al*. 2007). Constructing mechanistic models that capture the detail of these fluctuations is complicated by the array of effectors associated with the immune response, the abundance of feedbacks designed to keep potentially harmful immune responses in check (Graham, Allen & Read 2005), the complex role of host memory (Antia, Ganusov & Ahmed 2005) and the dynamic nature of parasite growth itself (Antia & Lipstich 1997). Examples of analytical models (generally built around partial differential equations) based on empirical data include models developed for HIV (Perelson 2002), influenza (Saenz *et al*. 2010) and malaria in murine (Haydon *et al*. 2003; Mideo *et al*. 2008) and human hosts (Molineaux & Dietz 1999). For these examples, considerable data and detailed biological knowledge are available, and models have further deepened our understanding of the processes driving the time course of infection. Nevertheless, model development and appropriate parameterization in the face of available data remains non‐trivial (nonlinear feedbacks result in extremely erratic likelihood surfaces, leading to ambiguity in parameter estimates); and further, efforts to extend these models to connect within‐host dynamics to population outcomes remain rare (Gog *et al*. 2014).

Linking individuals to population outcomes is of fundamental relevance for both ecological and evolutionary questions (Metcalf *et al*. 2014). Population‐scale questions such as the impact of coinfection on transmission (Graham *et al*. 2007), the spread of resistance mutations in the face of chemotherapy (Kouyos *et al*. 2014) or determinants of spillover, in terms of what makes populations viable reservoirs, (Brook & Dobson 2015) require cross‐scale models capable of capturing individual differences and integrating across them to evaluate population‐level outcomes.

Many of the variables that drive the key processes linking individuals to populations (transmission potential, host survival, etc.) have in common the fact that they are quantitative traits (e.g. concentrations of virions, unicellular parasites, antibodies and lymphocytes in the blood). Integral Projection Models (IPMs) are now broadly used in ecology and evolution to capture demographic outcomes linked to continuous individual‐level variables such as size (Easterling, Ellner & Dixon 2000; Childs *et al*. 2011; Merow *et al*. 2014). Focal variables generally reflect individual life‐history or physiological traits such as size, weight, height, snout to vent length and tarsus length. The dynamics of these traits (e.g. increases in size via growth or losses via shrinkage) and their links to survival or fertility are modelled using generalized linear regression approaches (Easterling, Ellner & Dixon 2000). A transition kernel reflecting these functions defines transitions between sizes (or other chosen traits) over a discrete time step, usually a year. In the simplest analysis, the structure of the transition kernel is broadly analogous to a classic matrix population model (Caswell 2001) with a diagonal reflecting transitions linked to growth and survival and another important transition area linking adult size to offspring size. The key difference is that rather than discrete probabilities describing how individuals in a particular stage might be distributed across the range of possible stages at the next time step, a density relates current size to the continuous distribution of future sizes.

One of the major strengths of the IPM approach is that their formulation via a probability density allows inclusion of variation in trajectories across individuals and through time. For evolutionary models of continuous traits such as size at flowering of monocarpic plants (Metcalf *et al*. 2008), or ecological models exploring the impact of changes in body size on population dynamics (Ozgul *et al*. 2010), capturing these details can be key. Selection on life histories, in particular, will be modified by individual variation in trajectories – for example, the variance in growth trajectories of individuals from the same genetic background decreases the optimal flowering size in monocarpic plants (Childs *et al*. 2003), and including individual variation this is therefore essential for inference.

In the context of infectious disease dynamics, an IPM framework can capture consistent individual differences as well as temporal fluctuations in antibody titre, other metrics of immunological activity and their downstream effects on within‐host–parasite abundance, which might result from varied nutritional status and/or history of infection. Furthermore, and importantly, the impact of individual trajectories on individual host‐level outcomes, such as survival, and resulting population‐level characteristics, such as transmission, can be appropriately reflected. IPMs have been deployed to explore transmission of fungal parasites across a size‐structured coral population (Ellner *et al*. 2007); the impact of fungal symbionts on the population growth rate of two grass species (Chung, Miller & Rudgers 2015), ant symbionts on the population growth rate of a cactus (Ford *et al*. 2015) and a variant has been used to explore heritability of set point viral loads (Bonhoeffer *et al*. 2015). All of these have yielded insights into the process of transmission, or the effect of the pathogen on population dynamics, but to date there has been relatively little work capitalizing on the strengths of IPMs in the context of within‐host dynamics and the population ecology of infectious diseases in mammals.

Here, we start by introducing the broad categories of infectious disease‐related traits that might be modelled using an IPM approach and some of the questions that could be tackled with such an approach and then move on to examine two case studies on murine malaria and maternal antibodies against measles; we conclude by discussing when an IPM approach might be most appropriate and key future directions.

## IPMs for host–parasite dynamics

Infectious disease dynamics inherently contain at least two species (the parasite and the host) – and this number is increased where multihost–parasites, or spillover from one host into another, are the focus. Building on developments in modelling complex life cycles with IPMs (Ellner & Rees 2006; Metcalf *et al*. 2013) and modelling infectious disease dynamics in structured populations (Klepac & Caswell 2011), it is straightforward to adapt an IPM framework to efficiently capture traits of hosts, parasites or the combined host–parasite dyad.

Parasites can be broadly categorized into two groups – *acute parasites*, associated with short duration infections; and *chronic parasites*, where infection is long lasting. Often, this distinction aligns with the distinction between microparasites (which multiply within the host) and macroparasites (e.g. many helminths, which do not). There are, of course, exceptions – microparasites such as HIV may result in chronic infections. However, for both acute and chronic parasites, at the within‐host scale, infection is an inherently dynamic process, reflected by changes in an array of continuous traits.

To start with acute infections, parasite multiplication results in increasing abundance and spread of the parasite within the host, but this is in turn mitigated by both target cell depletion (where target cells are the resources targeted by the parasite) and immune system activity (Graham 2008; Metcalf *et al*. 2011). As a result of these interactions, a focal trait such as parasite load will tend to rapidly increase and then decrease. Capturing this in an IPM framework will typically require abstracting the underlying dynamical details, as an IPM generally reflects broad features of trait changes using regression tools (but see Discussion for possible extensions). One option to appropriately capture the extreme changes observed over the course of an acute infection is to include a further structuring of traits along an axis such as day post‐infection (analogous to IPMs where individuals experience different transitions across size for each age class (Childs *et al*. 2011), they can experience different transitions across parasite load based on day post‐infection), illustrated below (Case study 1: Estimating probability of onward transmission from within‐host dynamics of malaria). However, care will be required in estimating and interpreting the underlying parameters, as many host–parasite IPMs will implicitly codify assumptions about host and parasite traits and their relationship. For example, for an IPM describing the dynamics of parasitaemia, the parameter reflecting the initial parasite dose could easily be increased to explore the effects on prevalence in the population. However, results will be misleading if the model does not explicitly include what is likely to be a nonlinear relationship between initial parasite dose and the resulting host immune response. Explicit codification could be achieved by defining a specific functional form that links dose dependence and immune induction in the model, but if data are not available with which to parameterize such a relationship (here, such data might include multiple starting doses), then the assumed functional form is necessarily speculative. This is, of course, a very general issue in model construction, but one which may be of particular importance here, as the formulation of within‐host dynamics (which are notoriously reactive) via regression tools may result in an array of cryptic assumptions being made, and special care should be taken to evaluating the importance of these. As a result, the strongest inference may follow from comparative analyses across different clones, pathogens or hosts rather than exploratory model perturbations and forward simulation – an example is provided in the malaria model described below. Nevertheless, in some cases, of course, interpretation of parameters linked to some aspects of host or parasite biology (e.g. host fertility, host mortality, parasite growth and parasite reproduction) may be straightforward and perturbation analyses may be a powerful direction for inference.

Parasite load is clearly not the only possible focal trait – for acute parasites, infection will usually lead to a rapid increase in immune activation, including the rate of proliferation of cells of the immune system, boosting of the signalling molecules that the cells secrete and production of antibodies specific to the parasite; all these processes are often subsequently rapidly downregulated to avoid immunopathology (Graham, Allen & Read 2005). An array of related continuous features could be modelled using an IPM‐like framework. The scope for the potential array of models here is enormous – the rate at which cells and molecules are induced and decay, and the degree to which they provide indicators of exposure vs. disease may be both parasite and effector specific. It is worth noting that some of the most successful infectious disease models deployed to date have been powerful exactly because immunity has such extreme dynamics that it effectively acts as a binary trait (i.e. for completely immunizing infections such as measles, Bjørnstad, Finkenstadt & Grenfell 2002), allowing the detail of within‐host dynamics to be ignored. Nevertheless, completely immunizing infections remain a relatively special case, and there is scope for investigating immune dynamics related to an array of other parasites where; for example, antibody titre provides a correlate of demographic outcomes, such as mortality, or infection probability, relevant to bridging between scales. Even in the case of immunizing infections, there are some areas where more subtle effects are expected – maternal immunity provides a special case of antibody dynamics for measles, discussed in detail below (Case study 2: Exploring dynamical consequences of individual variability in immune parameters: maternal immunity to measles).

Moving to the case of chronic infections, for macroparasites, such as helminths, the parasites may grow within the host, increasing depletion of host resources (Hayward *et al*. 2014b), but they do not generally increase in abundance (apart from ingestion of additional transmission stages such as eggs). More generally, for chronic parasites (which includes many macroparasites), in mathematical terms, we can often think of parasite density and the host's immune system (i.e. target cell production) as settling to equilibrium. The details of the dynamics leading up to equilibrium may be of less importance, and relative to acute infections, it is thus more straightforward to envisage capturing within‐host dynamics using regression tools, as the extreme fluctuations in focal traits such as those that characterize acute infections are avoided. Key continuous traits that might be modelled using an IPM framework for chronic parasites might include parasite load (Bonhoeffer *et al*. 2015), the density of parasite‐specific lymphocytes (Borchers *et al*. 2014) or parasite length in the case of helminths. Insights are likely to emerge from understanding either the trajectory to the equilibrium (where this is amenable to regression modelling), but also and perhaps more powerfully, the role of variance around the mean in parasite load, as was explored in a recent paper by Bonhoeffer *et al*. (2015). Approximating all populations and processes by Gaussian distributions, they derived analytical expressions to describe change in the distribution of HIV set point load over a single transmission cycle. In principle, their model could be reformulated as an IPM to allow greater flexibility in the underlying distributional assumptions.

Chronic and acute pathogens will also elicit responses from the immune system's enormously complex and frequently dynamic set of effectors. Some components may be relatively constant, putting the emphasis for IPM‐based inference more on capturing individual variance rather than capturing the details of the trajectories through time. For example, titres of self‐reactive antibody, a marker used to identify autoimmune diseases, differ markedly among individuals, but are consistent within individuals across time, thus providing a heterogeneous but static individual trait (Vindenes & Langangen 2015), in contrast to the dynamic traits described so far. In Soay sheep, self‐reactive antibodies correlate positively with survival and parasite‐specific antibody and negatively with annual fertility (Graham *et al*. 2010). Parasite‐specific antibodies provide additional power to predict survival of individuals (Nussey *et al*. 2014). With detailed data available to translate such variables into individual‐level consequences (e.g. linking antibody titres to host survival and fertility), an IPM approach could enable powerful exploration of the population‐level consequences of trait heterogeneity across individuals and the roles, for example, of alternative modes of defence (such as resistance vs. tolerance) at the epidemiological scale (Hayward *et al*. 2014a).

**Figure 1:**
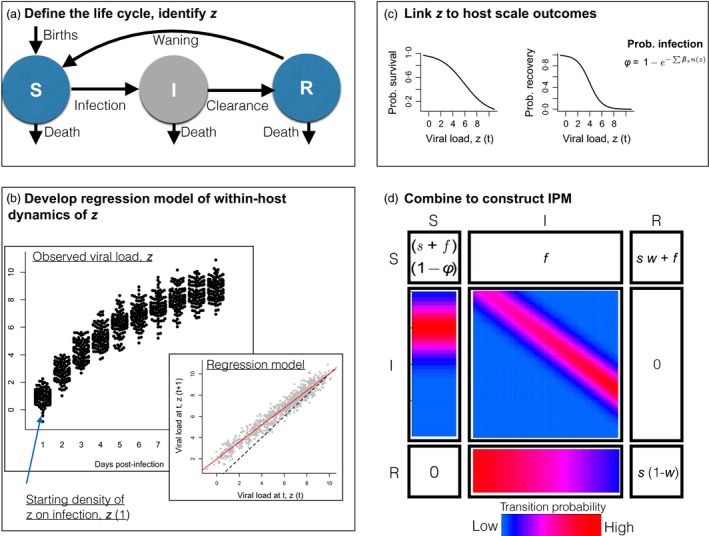
**Hypothetical example of the workflow for construction of an infectious disease IPM**. (a) Define the life cycle (here a classic SIR, or Susceptible‐Infected‐Recovered framework) and identify the continuous feature of within‐host dynamics that is the focal variable; here taken as viral load, and denoted by *z*; (b) Frame the observed dynamics of *z* (here shown as a function of days post‐infection on the *x* axis), to allow an appropriate regression model to be fitted. Here, the data is re‐plotted to relate *z* 1 day in the future to the current value of *z* (grey points), which is well described by a linear regression (line); the density of *z* on the first day of infection is also required to construct the IPM, and can be simply obtained by fitting a mean and variance to the distribution of *z* observed on day 1; (c) Use regression modelling to describe the relationship between *z* and key host level outcomes, here taken to be recovery and survival, and probability that a susceptible individual becomes infected, φ. The latter depends on the relationship between viral load *z* and transmission, β_*z*_ as well as *I*(*z*), the number of individuals with viral load *z*. Other relationships are, of course, possible (e.g. with fertility) but are not illustrated here. (d) Construct the associated IPM model, where the transition from susceptibility to infection depends on the density of viral load across the population, and is associated with a starting density of viral load, *z*; viral load evolves according to a linear regression over the course of the infection, and there is a probability of survival and recovery during infection associated with viral load. Other variables include survival of uninfected individuals, *s*; fertility, *f*; and the probability of waning of immunity, *w*.

For both chronic and acute infections, successful construction of IPMs, or any other models that bridge scales of biological processes, will require (i) modelling within‐host dynamics (using regression tools in the case of IPMs), (ii) careful interpretation of associated parameters and (iii) robust translation of the chosen focal trait into processes that have population‐level effects. Specifically, host traits such as recovery, survival or fertility (Fig. 1) will translate to the population level of disease dynamics, via their influence on transmission potential, which may also scale with individual viral load or other features of within‐host dynamics.

## Case study 1: Estimating probability of onward transmission from within‐host dynamics of malaria

**Figure 2:**
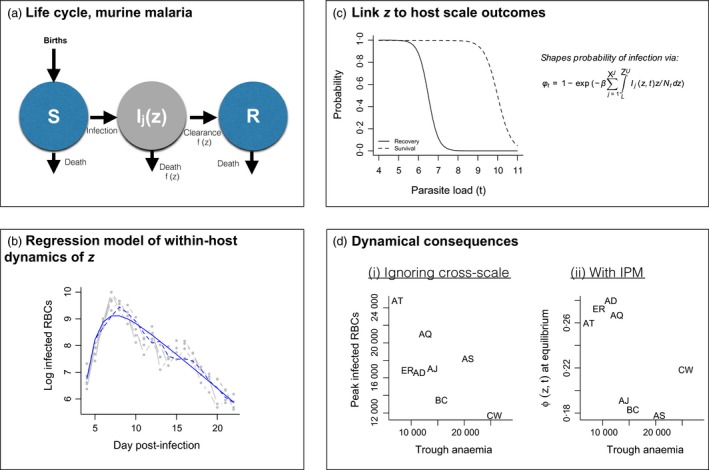
Constructing a model of murine malaria (following Fig. 1). (a) The life cycle, with choice of parasite density as *z*, a variable which shapes both the rate of recovery and survival (*f*(*z*) indicates a dependence on *z*); the susceptible (S), infected (I) and recovered (R) classes all contribute to births, not shown for clarity. (b) **Within‐host dynamics** showing the time series of parasite density in the blood phase of malaria for five mice infected with the AD clone (grey points) and fitted linear regression predicted log infected RBCs as a function of current burden and time step either one time step ahead (dashed line) or over the full time course (solid line); parameters are provided in Table 1. Similar patterns are obtained for the 7 other clones (Fig. S1). (c) **Host scale dynamics** including assumed patterns of recovery and survival as a function of the log infected RBC burden required to construct an IPM (see Table 1), and equation defining the probability of infection. (d) **Comparison of broad inference** (i) when ignoring cross‐scale dynamics, the maximal infected RBCs observed over the time course of infection across clones (*y*‐axis) are negatively associated with the depth of the trough of RBCs (*x*‐axis), an indicator of anaemia, a relationship attributed to a virulence‐transmission trade‐off (Metcalf *et al*. 2012); (ii) using an IPM to capture cross‐scale dynamics indicates that the probability of infection of susceptible individuals at equilibrium estimated from the full IPM (*y*‐axis) shows no clear relationship with the trough of RBCs (*x*‐axis), suggesting that the cross‐scale dynamics (incorporation of survival and recovery) dilute this effect.

In the bloodstream phase of malaria (*Plasmodium* spp.), infected red blood cells (RBCs) burst in synchrony, releasing merozoites that may infect a new RBC. Twenty‐four or forty‐eight hours later (depending on the malaria species), the next generation of merozoites bursts out, and the cycle then repeats itself until the host dies or clears the infection (Fig. 2a). Over the course of the infection, a fraction of the infected cells develop into sexual forms that are taken up by mosquitoes. When the mosquito bites its next host, the parasite migrates to the liver. Parasites then emerge from the liver, and the bloodstream phase starts again (Metcalf *et al*. 2012).

Here, to illustrate a population‐scale inference arising from IPM analysis of within‐host dynamics, we leverage data describing the bloodstream phase of a range of clones of rodent malaria (*P. chabaudi*, described in Long *et al*. 2008a,b; Metcalf *et al*. 2012) to explore the consequences of these within‐host dynamics for population‐level outcomes, specifically rates of onward transmission. We do so by nesting an IPM within a basic SIR population model. We model the within‐host dynamics of this acute infection by tracking the log number of infected RBCs as our focal trait, denoted *z*. In principle, it is also possible to use the number of infected RBCs directly as the focal trait, leading to a matrix population model parameterized with regression tools. However, the log scale is more practical since the range of RBCs spans several orders of magnitude and never approaches zero. Infected RBC load shapes host survival, recovery and transmission (Mackinnon & Read 2004b); and thus it is an appropriate focal trait. To also account for the dynamics of this acute 24 h cycling parasite over the course of the infection, we further structure our model by day post‐infection, ranging from 1 to *J* days post‐infection. The form of the population model is as follows:

$$\begin{array}{cc} S(t+1) & =sS\left(t\right)\left(1-\phi_{t}\right)+fS\left(t\right)+f\sum_{j=1}^{J}\int_{L}^{U}I_{j}\left(z,t\right)dz+fR\left(t\right)\\ I_{0}\left(z^{\prime },t+1\right) & =s\phi_{t}S\left(t\right)G_{0}\left(z^{\prime }\right)dz\\ I_{j+1}\left(z^{\prime },t+1\right) & =\int_{L}^{U}G_{j}\left(z^{\prime },z\right)s_{z}\left(z\right)\left(1-r_{j}\left(z\right)\right)I_{j}\left(z,t\right)dz\;\left[\text{for}\;j<\left(J-1\right)\right]\\ I_{J}\left(z^{\prime },t+1\right) & =\int_{L}^{U}G_{J-1}\left(z^{\prime },z\right)s_{z}\left(z\right)\left(1-r_{J-1}\left(z\right)\right)I_{J-1}\left(z,t\right)dz+\int_{L}^{U}G_{J}\left(z^{\prime },z\right)s_{z}\left(z\right)\left(1-r_{J}\left(z\right)\right)I_{J}\left(z,t\right)dz\\ R(t+1) & =\sum_{j=1}^{J}\int_{L}^{U}s_{z}\left(z\right)r_{j}\left(z\right)I_{j}\left(z,t\right)dz+sR\left(t\right) \end{array},$$

where *S*(*t*) indicates the number of susceptible individuals at time *t*,* I*_*j*_(*z,t*) captures the number of infected individuals at time *t* with log infected RBC load *z*, on day *j* post‐infection (and the prime in *I*_*j*_(*z',t + *1) indicates that this reflects the log infected RBC load the following time step), and *R*(*t*) is the number of recovered individuals at time *t*;* U* and *L* indicate the limits of integration across log infected RBC load, where the upper limit (*U*) reflects a value slightly larger than the maximum observed (which here reflects the maximum observed log infected RBC load) and likewise for the lower limit (*L*). Finally, J is the total number of days post‐infection modelled. Note that the discrete time unit for this compound IPM (compound because it is structured by both day post‐infection and log infected RBC load) is one day – it would also be possible to separate the time‐scales of the within‐host and between‐host dynamics (Heffernan & Keeling 2009), but we chose to keep them unified in the two case studies illustrated here. We model mouse fertility as a constant, captured by the parameter *f*, and survival as a constant captured by the parameter *s*, except in infected individuals whose survival is related to their parasite load *z,* as described by a probability *s*_*z*_(*z*). All of these parameters are adjusted to reflect 1 day to match the time‐scale of the within‐host infection process. The probability of infection is captured by ϕ_*t*_, further described below. Upon infection, the distribution of log infected RBCs that hosts experience is captured by the density function *G*_0_(*z*); subsequent transitions between infected states are captured by the kernel *G*_*j*_(*z'*,*z*); and the probability of recovery is described by *r*_*j*_(*z*); see Table 1 for details and functional forms.

**Table 1:** Parameters and functional forms for IPMs relating to murine malaria, indicating parameters for the AD clone, shown in Fig. 2a. The full array of parameters across clones is shown in Table S1 (Supporting information). For the numerical integration, we set the upper limit of integration *U* = 12, the lower limit *L* = 3 and the number of days post‐infection tracked to be *J* = 15

Description	Functional form	Parameters	Details of parameterization
Dynamics of log infected RBCs, *G*_*j*_(*z',z*)	*z*_*t*_ = *a*_*g*_ + *b*_*g*_ *z*_*t–*1_ + *c*_*g*_ *j* + *d*_*g*_ *j* ^2^ *+ *ε	*a*_*g*_ = 4*·*83*, b*_*g*_ = *−*0*·*11*, c*_*g*_ * *= 0·51, *d*_*g*_ = *−*0·0003*,* ε *= N* (0*,* σ *= *0·43)	Fitted to data
Starting density of *z* on infection, *G*_0_(*z'*)	*z*_0_ = *a*_0_ + ω	*a*_0_ = 6·75; ω = 0·37	Fitted to data
Survival of infected individuals	logit(*s*_*z*_(*x*)) = *m*_0_ + *m*_*s*_ *x*	*m*_0_ = 30, *m*_*s*_ = *−*3	Specified to result in mortality for mice with z>xx, based on previous analyses
Recovery of infected individuals	logit(*r*_*j*_(*x*)) = *r*_0_ + *r*_*s*_ *x*	*r*_0_ = 26, *r*_*s*_ * = −*4 *for j<J*	Specified to result in recovery if z falls below 7; and for j=J, in the absence of further information, we set r_j_=1
Survival of uninfected individuals	*s*	0·997	Based on an average mouse life span of 12 months
Fertility	*f*	0·13	Based on average mouse fertility of 5 litters of 10 mice per year
Transmission	β	5	Assuming approximately 5 new infections per infected individual.

The probability of infection of susceptible individuals, ϕ_*t*_, must encompass the density of the asexual form (captured here by log infected RBCs, *z*), allocation by the asexual blood phase to the sexual form, ingestion of this form by a mosquito and transmission to a new host. Although differences in allocation to sexual reproduction across clones and through time have been observed (Mackinnon & Read 1999; Long *et al*. 2008a,b; Metcalf *et al*. 2012), we initially ignore this complexity and assume that uptake of the parasite by mosquitoes scales with the numbers of infected RBCs; modelling transmission as a frequency‐dependent process then captures vector‐borne transmission. Assembling these elements, the probability of infection of a susceptible individual is defined by:

$$\phi_{t}=1-\text{exp}\left(-\beta \sum_{j=1}^{J}\int_{L}^{U}I_{j}\left(z,t\right)z/N_{t}dz\right),$$

where β captures the overall scaling of transmission and *N*_*t*_ is the total host population size at time *t*. For the purposes of this illustration, we assume that there is no waning of immunity. The time course of infection in one exemplar clone, the AS clone, and associated model fits are shown in Fig. 2a. The full set of 8 clones is shown in Fig. S1 (Supporting information). These clones have been characterized as falling along a virulence‐transmission trade‐off – the clones that result in the greatest anaemia also have the highest parasite loads, presumed to correlate with transmission (Metcalf *et al*. 2012). Such a pattern is an expected evolutionary outcome – both low and high virulence pathogens might achieve equivalent fitness and coexist if the fitness cost resulting from host mortality experienced by high virulence pathogens is offset by high rates of transmission (Anderson & May 1982). However, the degree to which within‐host patterns suggestive of a virulence‐transmission trade‐off translate into the population‐scale pattern that evolutionary predictions would suggest is unclear.

To evaluate outcomes at the population scale from this model, we first combine the regression models fit to the dynamics of log infected RBCs (Figs 2b, S1, Table 1) with assumptions about how the burden of infected RBCs affects survival and recovery probabilities (Fig. 2c), and then numerically integrate the resulting IPM using the ‘mid‐point rule’ (Rees, Childs & Ellner 2014). With this, we can project the population forwards and track densities of log infected RBCs (Fig. S2, Supporting information) as well as the process of infection.

Malaria has been held up as a powerful example of the virulence‐transmission trade‐off (Mackinnon & Read 2004b), and the set of clones available in these data illustrate this very clearly, with the clones that lead to the greatest anaemia also having the highest peaks of parasite density and thus, by inference, transmission potential (Fig. 2d). However, these patterns describe individual host‐level outcomes. The degree to which ‘transmission potential’ translates to population‐level outcomes such as incidence of infection is unclear (Alizon *et al*. 2009). Using our IPM framework, we can evaluate this across the eight clones presented here, comparing the probability that a susceptible individual will be infected at equilibrium, ϕ_*t*_, with the depth of the trough of anaemia for each clone (Fig. 2d). If the population‐scale outcomes simply reflect within‐host outcomes, we would expect that the most virulent clones (i.e. those associated with the greatest parasite density) are also the clones that pose the highest risk to susceptible individuals – that is a positive relationship. In fact, we find that the virulence‐transmission pattern expected is considerably diluted with the full population model – although virulent strains, for which the trough of RBCs is particularly deep, tend to have high ϕ_*t*_ at equilibrium, the pattern across the clones reverses the trade‐off, and the least virulent clone (CW) achieves substantially more transmission than within‐host pattern predicts. In other words, processes occurring across scales obscure the outcome we would expect from a virulence‐transmission trade‐off at the scale of the host individual – and in fact, one might conclude that the host‐level pattern is less an outcome of selection than a simple emergent property of the process of infection in malaria – inevitably, gain of one infected RBC requires loss of one uninfected RBC.

Intriguingly, these results also suggest that at the population‐level scale, the AS clone deviates from the broad positive relationship seen across the other clones and has a much lower risk of onward transmission to susceptible individuals, ϕ_*t*_. The AS clone has an especially long history of propagation through serial passage, which is expected to select for increased virulence (Mackinnon & Read 2004a) – this adaptation might diminish performance when a population‐level perspective is taken, as is suggested here (Fig. 2d). However, the many components of the model for which we did not introduce clone‐specific parameters (gametocyte production and, particularly, recovery) might either diminish or accentuate this result. This is an exciting area for future research.

There are a number of issues that should also be considered in evaluating the patterns reported, and which are of more general importance in considering the utility of IPMs for capturing within‐host dynamics. First, the time course available does not entirely resolve the full infection for the range of clones (Fig. S1) – in fact, many clones experience secondary increases before the end of the time course (recrudescence), and few mice have recovered – without detailed parameterization of this process, the IPM cannot capture it and therefore may under‐ or overestimate population‐level outcomes as a result. This is likely to be a very general consideration in both experimental and natural systems. Since the force of infection in the population will depend on transmission across individuals at all stages of the infection, the model will be sensitive to the exact choice of maximal days post‐infection modelled (*J*) as well as the parameterization of the recovery process. Furthermore, choice of insufficiently large *J* may result in heaping in the categories corresponding to the last day post‐infection modelled, or unintentional eviction from the IPM (see Williams, Miller & Ellner (2012) for more discussion of this issue). On the positive side, further sensitivity analysis (such as exploration of the consequences of parameter perturbation (Caswell 2001), albeit keeping in mind the caveats outlined above) with the IPM framework would allow evaluation of exactly how important these last infection processes are, and which are the key parameters for which further investment and investigation would be most beneficial. Sensitivity to survival, fertility and transmission parameters could likewise be explored.

## Case study 2: Exploring dynamical consequences of individual variability in immune parameters: maternal immunity to measles

Women who have been exposed to measles during their lifetime and developed antibodies to this parasite can transfer those antibodies to their offspring (Nicoara *et al*. 1999). Following birth, these transferred antibodies continue to protect the child from infection by measles. However, maternally transferred antibodies degrade over time, and once their concentration has waned to negligible levels, offspring are once again vulnerable to measles (Cáceres, Strebel & Sutter 2000). The exact magnitude of the transferred antibodies affects the time until susceptibility and can thus have population‐level consequences. Furthermore, vaccinated mothers are known to transfer lower levels of antibodies to their children, a phenomenon echoed by heterogeneities in transfer of maternal antibodies observed from birds to mammals (Boulinier & Staszewski 2008), and the dynamical consequences of this are still unresolved.

**Figure 3:**
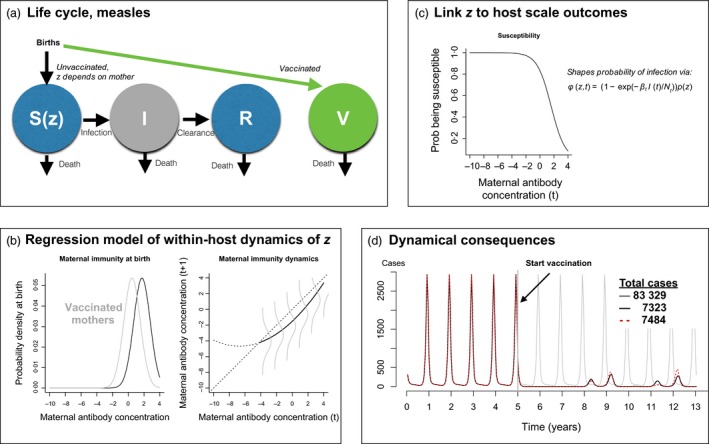
**Dynamical consequences of effects of vaccination on maternal antibodies (following Fig. 1)**. (a) **The life cycle**, with choice of maternal antibodies as *z*, a variable which shapes the loss of susceptibility and thus infection (*f*(*z*) indicates a dependence on *z*); the Susceptible (S), Infected (I) and Recovered (R) and Vaccinated (V) classes all contribute to births, and the level of *z* is defined by the identity of the mother, see text for details. (b) **Within‐host dynamics** showing the distribution of maternal antibodies at birth for unvaccinated (black) and vaccinated (grey) mothers, and their subsequent decline; where the distribution around this decline obtained via a regression model (grey lines); (c) **Host scale dynamics** including the probability of become susceptible as a function of maternal antibodies; see Table 2 for parameters and their sources. (d) Simulations from the IPM model showing cases obtained under a simulation with no vaccination (grey line) and cases obtained under 85% vaccination when mothers do (black line) or do not (dashed line) differ in their maternal immunity; indicating the counter‐intuitive outcome that increased protection of offspring can actually result in more cases via nonlinear effects. The legend indicates total cases after the start of vaccination in each of the three scenarios – both vaccination scenarios reduce the number of cases, but counter‐intuitively, if vaccinated mothers transfer antibody concentrations as high as naturally infected mothers, the total number of cases may be higher as a result of transient dynamics.

We use data and models presented in Waaijenborg *et al*. (2013) to develop regression models to capture the pattern of decline of maternal antibodies as a function of current levels (obtained by simulating from the model they develop to describe antibody concentration as a function of age), as well as initial densities and threshold marking the transition to susceptibility (Fig. 3a–c, Table 2). As above, we nest this within an SIR framework but now including a ‘*V*’ category for vaccinated individuals:

$$\begin{array}{cc} S(z^{\prime }t+1) & =\left(1-\nu \right)\left[A_{0}\left(z^{\prime }\right)B+A_{0\nu }\left(z^{\prime }\right)B\nu \right]+s\int_{L}^{U}A\left(z^{\prime },z\right)\left(1-\phi \left(z,t\right)\right)S\left(z,t\right)\;dz\\ I(t+1) & =s\int_{L}^{U}A\left(z^{\prime },z\right)\phi \left(z,t\right)S\left(z,t\right)dz\\ R(t+1) & =sR(t)+sI(t)\\ V(t+1) & =\nu \left[B+B_{\nu }\right]+sV\left(t\right) \end{array},$$

where *S*(*z*,*t*) reflects the number of susceptible individuals with maternal antibody concentration z, *I*(*t*) is the number of infected individuals, *R*(*t*) is the number of recovered (and completely immune) individuals, and *V*(*t*) is the number of vaccinated individuals (also completely immune). We could also have chosen to model an explicit maternally immune or ‘M’ compartment, but instead chose to consider maternal antibodies as indicative of susceptibility status for simplicity. The time step here taken as 2 weeks, which reflects the approximate generation time of measles (Grenfell, Bjornstad & Finkenstädt 2002). The probability of vaccination in one‐two‐week time step is captured by *v*;* s* is the probability of survival (we ignore infection‐related mortality for simplicity), *f* is fertility, likewise, and ϕ(*z,t*) is the probability of infection of an individual with maternal antibody concentration *z* (further detailed below). At birth, the distribution of maternal antibody concentrations in infants is captured by the density function *A*_0_(z) for unvaccinated mothers and *A*_0*v*_(*z*) for vaccinated mothers, where *B* indicates the number of children born to unvaccinated mothers and *B*_*v*_ children born to vaccinated mothers; subsequent decline of maternal antibodies is captured by the kernel *A*(*z'*,*z*); the integration occurs between an upper and lower limit of antibody concentrations *U* and *L*, and recovery is complete within one‐two‐week period, so all infected individuals moved into the recovered stage at *t* + 1; see Table 2 for details of parameters and functional forms. The probability of infection, ϕ(*z,t*) is defined by

$$\phi \left(z,t\right)=(1-\text{exp}\left(-\beta_{t}I\left(t\right)/N_{t}\right))p\left(z\right),$$

where β_*t*_ captures seasonally varying transmission, *N*_*t*_ is the total host population size at time t, included as immunizing childhood infections generally scale in a frequency‐dependent fashion (Grenfell, Bjornstad & Finkenstädt 2002), and *p*(*z*) reflects the probability of being susceptible, for an individuals with antibody concentration *z*.

**Table 2:** Parameters and functional forms for IPMs relating to maternal antibodies of measles. For the numerical integration, we set the lower limit of integration *L* = *−*10 and the upper limit *U* = 4, based on data from Waaijenborg *et al*. (2013)

Description	Functional form	Parameters	Details of parameterization
Dynamics of waning of log maternal antibody concentration, *z*,* A*(*z'*,*z*)	*m*_*t*_ = *a*_*g*_ + *b*_*g*_ *m*_*t–*1_ + *c*_*g*_ *m* ^2^_*t–*1_ + ε	*a*_*g*_ = (*−*1·52*, b*_*g*_ = 0·94, *c*_*g*_ = 0·07 ε *= N* (0, σ = 1·28)	Fitted to a simulation based on data described in Fig. 1 of Waaijenborg *et al*. (2013); and parameters provided in the Table S1
Starting density of z for children born to infected mothers, *A*_0_(*z'*)	*z*_0*s*_ = *a*_0*s*_ + ω	*a*_0*s*_ = 1·74; ω_*s*_ = 1·11	Obtained from Waaijenborg *et al*. (2013)
Starting density of *z* for children born to vaccinated mothers, *A*_0v_(*z'*)	*z*_0*v*_ = *a*_0*v*_ + ω	*a*_0*v*_ = 0·48; ω_*s*_ = 1·11	Obtained from Waaijenborg *et al*. (2013)
Probability of being susceptible as a function of log maternal antibody concentration	logit(*s*_*s*_(*z*)) = *s*_0_ + *s*_1_ *z*	*s*_0_ = 1·60; *s*_*s*_ = *−*1	Based on a cut‐off titre of 1·60 for susceptibility (Waaijenborg *et al*. 2013)
Survival of uninfected individuals	*s*	0·99	Reflecting high biweekly survival
Total offspring each biweek	*B*	500	Reflecting a high fertility context
Transmission	β	18	Based on average *R*_0_ for measles
Seasonal forcing function (*t* indexes biweek in the simulation)	(1 + α cos(2π(*t/*24)))	α *= *0·5	Based on observed patterns for measles

To illustrate the use of IPMs in this setting, we simulated two contrasting situations – in both, vaccination was introduced, but in the first case, the observed difference between the offspring of vaccinated and unvaccinated mothers was implemented, and in the second, we assumed that this difference did not exist, that is *a*_0*v*_ **= *a*_0*s*_ (Table 2, Fig. 3d). Intriguingly, if vaccinated mothers supply their children with just as high a concentration of antibodies as unvaccinated mothers, this can nevertheless actually result in a higher burden of cases. This illustrates the importance of nonlinear feedbacks that characterize infectious disease dynamics – in this example, specifically, the effect of ‘honeymoons’ resulting from vaccination (McLean & Anderson 1988). Classically, this occurs because introduction of vaccination reduces transmission, thus leaving individual who are unvaccinated also unexposed to natural infection, allowing accumulation of susceptible individuals, potentially eventually resulting in a large outbreak. Here, the twist is that the absence of maternal protection in vaccinated individuals prevents the longer build‐ups of susceptible individuals, by accelerating waning of immunity, and allowing earlier, and thus smaller outbreaks. Removing this difference has the opposite effect – amplifying the honeymoon effect. By extension, it can clearly be seen that individual differences such as those described here linked to vaccination can have population‐level dynamical consequences. Further evaluation of the effects of individual heterogeneity as well as the trajectory of waning of immunity could be undertaken with the framework described here.

## Discussion

The overview we present here, in conjunction with the two case studies, suggests that there is considerable potential for using IPMs to understand the consequences of continuous traits for infectious disease dynamics, while also highlighting some of the technical challenges. These include all the usual issues experienced in IPM development (Williams, Miller & Ellner 2012), as well as some of the more subtle issues in interpretation and model construction, such as issues of extrapolating the regressions beyond the range of the data (Merow *et al*. 2014).

For any particularly study system, a further important consideration in evaluating the value of an IPM approach is the quality and characteristics of the data available. Longitudinal data that capture individual trajectories in the focal trait (such as pathogen load or antibody titre), as well as variance across individuals, are essential. Data that capture the full time course of the infection process will also be key, as appropriately detailing the process of recovery (or not) will be essential for capturing dynamics (see Case study 1: Estimating probability of onward transmission from within‐host dynamics of malaria). In the absence of such data, inverse modelling approaches where population‐scale data, such as relative abundance of individuals of different sizes, or prevalence of the infection, are used to strengthen inference (e.g. Cropper & Anderson 2004) may provide some power, but parameter identifiability is likely to be a major challenge. Broader data on the ecology and life history of the species will also be important for robust prediction. For example, seasonal birth pulses shape the ecology of many rodent species and may have profound effects on disease dynamics (Peel *et al*. 2014). It is straightforward to extend the models described here to incorporate such ecological realism. A particularly interesting dimension may be interaction between host ‘condition’ (often captured by some measure of body mass) and parasite or immune dynamics. Host condition may be influenced by disease burden and past environmental conditions. Since infection risk may in turn depend on the condition of individual hosts (Koski & Scott 2001; Beldomenico & Begon 2010), there is potential for feedbacks between the transmission processes and host trait dynamics, leading to complex both ecological and evolutionary dynamics (Boots *et al*. 2009; Hayward *et al*. 2014a).

Our focus in the two case studies presented has been on traits that reflect dynamical heterogeneity (Vindenes & Langangen 2015), which various lines of evidence suggest may be key to understanding ecological and evolutionary processes. For example, analyses of reproductive timing in a monocarpic perennial herb have shown that among‐individual variation in growth trajectories influences the age distribution of reproductive timing and that the costs and benefits of this variation are influenced by (among other factors) temporal variation in the mean annual growth rate (Childs *et al*. 2004; Rees *et al*. 2004). There has also been a recent expansion of analyses of the consequences of static heterogeneity (Vindenes & Langangen 2015), that is traits that vary across individuals, but that an individual will retain for its entire life. Failure to include such static heterogeneity into models has been shown to result in misestimation of both the long‐term population growth rate, but also an array of other key life‐history variables such as the mean age of mothers (Vindenes & Langangen 2015). Considering such features is likely to be of particular importance in models capturing infectious diseases, as there is considerable evidence for strong genetic or early environment signatures on responses to infection (Hill 1998).

The default tool for modelling infectious disease dynamics in structured populations has long been partial differential equations (Anderson & May 1991). The two case studies presented here suggest that IPMs might provide a tractable, data‐driven alternative. However, the degree to which IPMs are applicable for epidemiological systems will depend very much on the scale of data available – if data only exists at time steps that prove too coarse for regression‐based tools to capture the detail of within‐host dynamics, then IPMs are unlikely to be the best modelling approach. A major challenge to deploying IPMs for questions in the ecology and evolution of infectious disease may therefore be that the level of fine‐scale detail explored here is likely to be only rarely available, especially in field systems. On the other hand, IPMs might offer a framework for more theoretical investigations – for example, allowing investigation of specific functional forms and their effects on disease dynamics, providing a framework for generating hypotheses.

An array of powerful tools have been developed for matrix population models (Caswell 2001; Klepac & Caswell 2011) and extended for IPMs (e.g. Ellner & Rees 2007), opening the way to evaluating the impact of an array of important features such as stochasticity on disease dynamics in an IPM framework. There are also a number of open directions for interesting technical developments – for example, building on innovations linking age and stage with infectious disease dynamics in a matrix framework (Klepac & Caswell 2011), it should be possible to derive descriptors of the expected number of secondary infections depending on the underlying initial parasite burden (e.g. *R*_0_(*z*)). This would provide interesting ways to quantify the impact of individuals with underlying focal trait value *x* on the dynamics of infection, a statistically rooted way of tackling the role of superspreaders (Lloyd‐Smith *et al*. 2005).

The two case studies we provide both use phenomenological models to capture the complexity of feedback‐driven dynamics of the process of infection, thereby providing an abstraction of the true dynamics (feedbacks between immunity and parasitaemia, for instance). For maternal antibodies, where the process is unlikely to respond to perturbations such as infection (Cáceres, Strebel & Sutter 2000), this strategy is likely to be relatively robust. In the case of murine malaria, blood stage dynamics essentially reflect an SIR process themselves (Mideo *et al*. 2008; Metcalf *et al*. 2011, 2012), and careful interpretation of model predictions given the potential for unpredictable feedbacks is essential. While the phenomenological approach described is likely to be powerful for taking a comparative perspective, as illustrated above using clones with varying levels of virulence (populations, or environments could be similarly deployed), it precludes more dynamical investigations of the outcome of perturbations. For example, with the phenomenological models we develop, exploring the impact of increasing the rate of growth of the infected RBC population is impossible, as there is nothing in the model to describe how increased parasite load will increase immune activity. However, in principle, we could easily embed a further SIR within the host‐level SIR to capture the details of within‐host dynamics, aligning, for example, infected cell survival with host immune activity, as our response variable. This would enable fine‐scale analysis of the evolutionary consequences of changes in within‐host traits, such as allocation towards sexual reproduction and its fluctuations through time (Mideo & Day 2009), or synchrony in bursting of red blood cells (Mideo *et al*. 2013; Greischar, Read & Bjørnstad 2014).

To conclude, using IPMs to bring continuous traits to infectious disease models has the potential to provide a powerful new approach to tackling an array of important questions in animal ecology and evolution. As illustrated here with the rodent malaria example, leveraging a breadth of data to develop phenomenological models capturing interactions between underlying mechanistic processes can yield comparative insights into cross‐scale dynamics. For models, or model components, where the mechanisms are more directly reflected, perturbation analyses will also allow for exciting ecological and evolutionary developments. For example, explorations of the evolutionary dynamics of pathogen load, using approaches analogous to those used to explore timing of flowering of monocarpic plants (Metcalf *et al*. 2008) could generate new, and importantly, empirically grounded insights into virulence‐transmission trade‐offs. The density and/or frequency dependence of infectious disease dynamics could result in hard to predict evolutionary feedbacks linked to this trait, which might be captured using adaptive dynamic approaches (Dieckmann 1997). At a more ecological scale, the feedbacks inherent to infectious disease dynamics can lead to non‐intuitive outcomes, making models key to generating expectations; however, this also leads to highly sensitive dynamics, with complex transients, which can make empirically rooted models essential to predicting short‐term outcomes of underlying continuous traits. Overall, this is a promising area of research and one in which we expect an array of technical developments in coming years.

## Supporting information


**Figure S1.** The time series of parasitaemia in the blood phase of malaria for five mice infected with 8 different clones, clone names shown as plot titles, and fitted linear regression predicted log infected RBCs as a function of current burden and time‐step either one time‐step ahead (dashed line) or over the full time course (solid line).
**Figure S2.** The modelled density of log infected RBCs through time for each of the clones in the IPM. Earlier time‐steps are indicated by red colors, moving through to blue/purple for the final time‐steps – the density starts with a low mean log infected RBCs, which moves up, briefly, and then down.
**Table S1.** Full set of parameters across all murine malaria clones (column headings) obtained from the regression models fitted to the time course of parasite density; see Table 1 for parameters common across clones.

## References

[jane12456-bib-0001] Alizon, S. , Hurford, A. , Mideo, N. & Van Baalen, M. (2009) Virulence evolution and the trade‐off hypothesis: history, current state of affairs and the future. Journal of Evolutionary Biology, 22, 245–259. 19196383 10.1111/j.1420-9101.2008.01658.x

[jane12456-bib-0002] Anderson, R.M. & May, R.M. (1982) Coevolution of hosts and parasites. Parasitology, 85, 411–426. 6755367 10.1017/s0031182000055360

[jane12456-bib-0003] Anderson, R.M. & May, R.M. (1991) Infectious Diseases of Humans. Oxford University Press, Oxford, UK.

[jane12456-bib-0004] Antia, R. , Ganusov, V.V. & Ahmed, R. (2005) The roles of models in understanding CD8 + T‐cell memory. Nature Reviews Immunology, 5, 101–111. 10.1038/nri1550 15662368

[jane12456-bib-0005] Antia, R. & Lipstich, M. (1997) Mathematical models of parasite responses to host immune defenses. Parasitology, 115, S115–S167. 10.1017/s003118209700200x 9571700

[jane12456-bib-0006] Beldomenico, P.M. & Begon, M. (2010) Disease spread, susceptibility and infection intensity: vicious circles? Trends in Ecology & Evolution, 25, 21–27. 19782425 10.1016/j.tree.2009.06.015

[jane12456-bib-0007] Bjørnstad, O.N. , Finkenstadt, B. & Grenfell, B.T. (2002) Endemic and epidemic dynamics of measles: estimating epidemiological scaling with a time series SIR model. Ecological Monographs, 72, 169–184.

[jane12456-bib-0008] Bonhoeffer, S. , Fraser, C. , Leventhal, G.E. & Swanstrom, R. (2015) High heritability is compatible with the broad distribution of set point viral load in HIV carriers. PLoS Pathogens, 11, e1004634. 25658741 10.1371/journal.ppat.1004634 PMC4450065

[jane12456-bib-0009] Boots, M. , Best, A. , Miller, M.R. & White, A. (2009) The role of ecological feedbacks in the evolution of host defence: what does theory tell us? Philosophical Transactions of the Royal Society B: Biological Sciences, 364, 27–36. 10.1098/rstb.2008.0160 PMC2666696 18930880

[jane12456-bib-0010] Borchers, S. , Ogonek, J. , Varanasi, P.R. , Tischer, S. , Bremm, M. , Eiz‐Vesper, B. *et al* (2014) Multimer monitoring of CMV‐specific T cells in research and in clinical applications. Diagnostic Microbiology and Infectious Disease, 78, 201–212. 24331953 10.1016/j.diagmicrobio.2013.11.007

[jane12456-bib-0011] Boulinier, T. & Staszewski, V. (2008) Maternal transfer of antibodies: raising immuno‐ecology issues. Trends in Ecology & Evolution, 23, 282–288. 18375011 10.1016/j.tree.2007.12.006

[jane12456-bib-0012] Brook, C.E. & Dobson, A.P. (2015) Bats as ‘special’ reservoirs for emerging zoonotic pathogens. Trends in Microbiology, 23, 172–180. 25572882 10.1016/j.tim.2014.12.004 PMC7126622

[jane12456-bib-0013] Cáceres, V.M. , Strebel, P.M. & Sutter, R.W. (2000) Factors determining prevalence of maternal antibody to measles virus throughout infancy: a review. Clinical Infectious Diseases, 31, 110–119. 10913406 10.1086/313926

[jane12456-bib-0014] Caswell, H. (2001) Matrix population models. Wiley Online Library.

[jane12456-bib-0015] Childs, D.Z. , Rees, M. , Rose, K.E. , Grubb, P.J. & Ellner, S.P. (2003) Evolution of complex flowering strategies: an age–and size–structured integral projection model. Proceedings of the Royal Society of London B: Biological Sciences, 270, 1829–1838. 10.1098/rspb.2003.2399 PMC1691450 12964986

[jane12456-bib-0016] Childs, D.Z. , Rees, M. , Rose, K.E. , Grubb, P.J. & Ellner, S.P. (2004) Evolution of size–dependent flowering in a variable environment: construction and analysis of a stochastic integral projection model. Proceedings of the Royal Society of London B: Biological Sciences, 271, 425–434. 10.1098/rspb.2003.2597 PMC1691612 15101702

[jane12456-bib-0017] Childs, D. , Coulson, T. , Pemberton, J. , Clutton‐Brock, T. & Rees, M. (2011) Predicting trait values and measuring selection in complex life histories: reproductive allocation decisions in Soay sheep. Ecology Letters, 14, 985–992. 21790931 10.1111/j.1461-0248.2011.01657.x

[jane12456-bib-0018] Chung, Y.A. , Miller, T.E. & Rudgers, J.A. (2015) Fungal symbionts maintain a rare plant population but demographic advantage drives the dominance of a common host. Journal of Ecology, 103, 967–977.

[jane12456-bib-0019] Cropper, W.P. & Anderson, P.J. (2004) Population dynamics of a tropical palm: use of a genetic algorithm for inverse parameter estimation. Ecological Modelling, 177, 119–127.

[jane12456-bib-0020] Dieckmann, U. (1997) Can adaptive dynamics invade? Trends in Ecology & Evolution, 12, 128–131. 21238006 10.1016/s0169-5347(97)01004-5

[jane12456-bib-0021] Easterling, M.R. , Ellner, S.P. & Dixon, P.M. (2000) Size‐specific sensitivity: applying a new structured population model. Ecology, 81, 694–708.

[jane12456-bib-0022] Ellner, S.P. & Rees, M. (2006) Integral projection models for species with complex demography. The American Naturalist, 167, 410–428. 10.1086/499438 16673349

[jane12456-bib-0023] Ellner, S.P. & Rees, M. (2007) Stochastic stable population growth in integral projection models: theory and application. Journal of Mathematical Biology, 54, 227–256. 17123085 10.1007/s00285-006-0044-8

[jane12456-bib-0024] Ellner, S.P. , Jones, E. , Mydlarz, L.D. & Harvell, C.D. (2007) Within‐host disease ecology in the sea fan *Gorgonia ventalina*: modeling the spatial immunodynamics of a coral‐pathogen interaction. American Naturalist, 170, E143–E161. 10.1086/522841 18171161

[jane12456-bib-0025] Ford, K.R. , Ness, J.H. , Bronstein, J.L. & Morris, W.F. (2015) The demographic consequences of mutualism: ants increase host‐plant fruit production but not population growth. Oecologia, 00, 1–12. 10.1007/s00442-015-3341-3 26003308

[jane12456-bib-0026] Gilchrist, M.A. , Coombs, D. & Perelson, A.S. (2004) Optimizing within‐host viral fitness: infected cell lifespan and virion production rate. Journal of Theoretical Biology, 229, 281–288. 15207481 10.1016/j.jtbi.2004.04.015

[jane12456-bib-0027] Gog, J.R. , Pellis, L. , Wood, J.L. , McLean, A.R. , Arinaminpathy, N. & Lloyd‐Smith, J.O. (2014) Seven challenges in modeling pathogen dynamics within‐host and across scales. Epidemics, 10, 45–48. 25843382 10.1016/j.epidem.2014.09.009

[jane12456-bib-0028] Graham, A.L. (2008) Ecological rules governing helminth‐microparasite co‐infection. Proceedings of the National Academy of Sciences of the United States of America, 105, 566–570. 18182496 10.1073/pnas.0707221105 PMC2206576

[jane12456-bib-0029] Graham, A.L. , Allen, J.E. & Read, A.F. (2005) Evolutionary causes and consequences of immunopathology. Annual Review of Ecology, Evolution, and Systematics, 00, 373–397.

[jane12456-bib-0030] Graham, A.L. , Cattadori, I.M. , Lloyd‐Smith, J.O. , Ferrari, M.J. & Bjørnstad, O.N. (2007) Transmission consequences of coinfection: cytokines writ large? Trends in Parasitology, 23, 284–291. 17466597 10.1016/j.pt.2007.04.005

[jane12456-bib-0031] Graham, A.L. , Hayward, A.D. , Watt, K.A. , Pilkington, J.G. , Pemberton, J.M. & Nussey, D.H. (2010) Fitness correlates of heritable variation in antibody responsiveness in a wild mammal. Science, 330, 662–665. 21030656 10.1126/science.1194878

[jane12456-bib-0032] Greischar, M.A. , Read, A.F. & Bjørnstad, O.N. (2014) Synchrony in malaria infections: how intensifying within‐host competition can be adaptive. The American Naturalist, 183, E36–E49. 10.1086/674357 PMC4334120 24464205

[jane12456-bib-0033] Grenfell, B.T. , Bjornstad, O.N. & Finkenstädt, B.F. (2002) Dynamics of measles epidemics: scaling noise, determinism, and predictability with the TSIR model. Ecological Monographs, 72, 185–202.

[jane12456-bib-0034] Haydon, D.T. , Matthews, L. , Timms, R. & Colegrave, N. (2003) Top‐down or bottom‐up regulation of intra‐host blood‐stage malaria: do malaria parasites most resemble the dynamics of prey or predator? Proceedings of the Royal Society of London Series B: Biological Sciences, 270, 289–298. 12614579 10.1098/rspb.2002.2203 PMC1691233

[jane12456-bib-0035] Hayward, A.D. , Garnier, R. , Watt, K.A. , Pilkington, J.G. , Grenfell, B.T. , Matthews, J.B. *et al* (2014a) Heritable, heterogeneous, and costly resistance of sheep against nematodes and potential feedbacks to epidemiological dynamics*. The American Naturalist, 184, S58–S76. 10.1086/676929 25061678

[jane12456-bib-0036] Hayward, A.D. , Nussey, D.H. , Wilson, A.J. , Berenos, C. , Pilkington, J.G. , Watt, K.A. *et al* (2014b) Natural selection on individual variation in tolerance of gastrointestinal nematode infection. PloS Biology, 12, e1001917. 25072883 10.1371/journal.pbio.1001917 PMC4114752

[jane12456-bib-0037] Heffernan, J. & Keeling, M.J. (2009) Implications of vaccination and waning immunity. Proceedings of the Royal Society of London B: Biological Sciences, 276, 2071–2080. 10.1098/rspb.2009.0057 PMC2677258 19324753

[jane12456-bib-0038] Hill, A.V. (1998) The immunogenetics of human infectious diseases. Annual Review of Immunology, 16, 593–617. 10.1146/annurev.immunol.16.1.593 9597143

[jane12456-bib-0039] Klepac, P. & Caswell, H. (2011) The stage‐structured epidemic: linking disease and demography with a multi‐state matrix approach model. Theoretical Ecology, 4, 301–319.

[jane12456-bib-0040] Koski, K.G. & Scott, M.E. (2001) Gastrointestinal nematodes, nutrition and immunity: breaking the negative spiral. Annual Review of Nutrition, 21, 297–321. 10.1146/annurev.nutr.21.1.297 11375439

[jane12456-bib-0041] Kouyos, R.D. , Metcalf, C.J.E. , Birger, R. , Klein, E.Y. , Zur Wiesch, P.A. , Ankomah, P. *et al* (2014) The path of least resistance: aggressive or moderate treatment? Proceedings of the Royal Society B: Biological Sciences, 281, 20140566. 25253451 10.1098/rspb.2014.0566 PMC4211439

[jane12456-bib-0042] Lloyd‐Smith, J.O. , Schreiber, S.J. , Kopp, P.E. & Getz, W.M. (2005) Superspreading and the effect of individual variation on disease emergence. Nature, 438, 355–359. 16292310 10.1038/nature04153 PMC7094981

[jane12456-bib-0043] Long, G.H. , Chan, B.H. , Allen, J.E. , Read, A.F. & Graham, A.L. (2008a) Blockade of TNF receptor 1 reduces disease severity but increases parasite transmission during *Plasmodium chabaudi chabaudi* infection. International Journal for Parasitology, 38, 1073–1081. 18226816 10.1016/j.ijpara.2007.12.001

[jane12456-bib-0044] Long, G.H. , Chan, B.H.K. , Allen, J.E. , Read, A.F. & Graham, A.L. (2008b) Experimental manipulation of immune‐mediated disease and its fitness costs for rodent malaria parasites. BMC Evolutionary Biology, 8, 128. 18447949 10.1186/1471-2148-8-128 PMC2391164

[jane12456-bib-0045] Mackinnon, M.J. & Read, A.F. (1999) Genetic relationships between parasite virulence and transmission in the rodent malaria *Plasmodium chabaudi* . Evolution, 53, 689–703. 10.1111/j.1558-5646.1999.tb05364.x 28565637

[jane12456-bib-0046] Mackinnon, M.J. & Read, A.F. (2004a) Immunity promotes virulence evolution in a malaria model. PloS Biology, 2, e230. 15221031 10.1371/journal.pbio.0020230 PMC434153

[jane12456-bib-0047] Mackinnon, M.J. & Read, A.F. (2004b) Virulence in malaria: an evolutionary viewpoint. Philosophical Transactions of the Royal Society of London. Series B, Biological Sciences, 359, 965–986. 15306410 10.1098/rstb.2003.1414 PMC1693375

[jane12456-bib-0048] McLean, A. & Anderson, R. (1988) Measles in developing countries. Part II. The predicted impact of mass vaccination. Epidemiology and Infection, 100, 419–442. 3378585 10.1017/s0950268800067170 PMC2249353

[jane12456-bib-0049] Merow, C. , Dahlgren, J.P. , Metcalf, C.J.E. , Childs, D.Z. , Evans, M.E. , Jongejans, E. *et al* (2014) Advancing population ecology with integral projection models: a practical guide. Methods in Ecology and Evolution, 5, 99–110.

[jane12456-bib-0050] Metcalf, C. , Rose, K. , Childs, D. , Sheppard, A. , Grubb, P. & Rees, M. (2008) Evolution of flowering decisions in a stochastic, density‐dependent environment. Proceedings of the National Academy of Sciences of the United States of America, 105, 10466–10470. 18641119 10.1073/pnas.0800777105 PMC2492471

[jane12456-bib-0051] Metcalf, C.J.E. , Graham, A.L. , Huijben, S. , Barclay, V.C. , Long, G.H. , Grenfell, B.T. *et al* (2011) Partitioning regulatory mechanisms of within‐host malaria dynamics using the effective propagation number. Science, 333, 984–988. 21852493 10.1126/science.1204588 PMC3891600

[jane12456-bib-0052] Metcalf, C. , Long, G. , Mideo, N. , Forester, J. , Bjørnstad, O. & Graham, A. (2012) Revealing mechanisms underlying variation in malaria virulence: effective propagation and host control of uninfected red blood cell supply. Journal of the Royal Society Interface, 9, 2804–2813. 10.1098/rsif.2012.0340 PMC3479917 22718989

[jane12456-bib-0053] Metcalf, C.J.E. , McMahon, S.M. , Salguero‐Gómez, R. & Jongejans, E. (2013) IPMpack: an R package for integral projection models. Methods in Ecology and Evolution, 4, 195–200.

[jane12456-bib-0054] Metcalf, C. , Birger, R. , Funk, S. , Kouyos, R. , Lloyd‐Smith, J. & Jansen, V. (2014) Five challenges in evolution and infectious diseases. Epidemics, 10, 40–44. 25843381 10.1016/j.epidem.2014.12.003

[jane12456-bib-0055] Metcalf, C.J.E. , Graham, A.L. , Martinez‐Bakker, M. & Childs, D.Z. (2016) Data from: opportunities and challenges of Integral Projection Models for modelling host‐parasite dynamics. Dryad Digital Repository, http://dx.doi.org/10.5061/dryad.07mc1. 10.1111/1365-2656.12456 PMC4991293 26620440

[jane12456-bib-0056] Mideo, N. & Day, T. (2009) On the evolution of reproductive restraint in malaria. Proceedings of the Royal Society of London ‐ Series B, 275, 1217–1224. 18303001 10.1098/rspb.2007.1545 PMC2602685

[jane12456-bib-0057] Mideo, N. , Barclay, V.C. , Chan, B.H.K. , Savill, N.J. , Read, A.F. & Day, T. (2008) Understanding and predicting strain‐specific patterns of pathogenesis in the rodent malaria *Plasmodium chabaudi* . American Naturalist, 172, E214–E238. 10.1086/591684 18834302

[jane12456-bib-0058] Mideo, N. , Reece, S.E. , Smith, A.L. & Metcalf, C.J.E. (2013) The Cinderella syndrome: why do malaria‐infected cells burst at midnight? Trends in Parasitology, 29, 10–16. 23253515 10.1016/j.pt.2012.10.006 PMC3925801

[jane12456-bib-0059] Molineaux, L. & Dietz, K. (1999) Review of intra‐host models of malaria. Parassitologia, 41, 221–231. 10697860

[jane12456-bib-0060] Nicoara, C. , Zach, K. , Trachsel, D. , Germann, D. & Matter, L. (1999) Decay of passively acquired maternal antibodies against measles, mumps and rubella viruses. Clinical and Diagnostic Laboratory Immunology, 6, 868–871. 10548578 10.1128/cdli.6.6.868-871.1999 PMC95790

[jane12456-bib-0061] Nussey, D.H. , Watt, K.A. , Clark, A. , Pilkington, J.G. , Pemberton, J.M. , Graham, A.L. *et al* (2014) Multivariate immune defences and fitness in the wild: complex but ecologically important associations among plasma antibodies, health and survival. Proceedings of the Royal Society B: Biological Sciences, 281, 20132931. 24500168 10.1098/rspb.2013.2931 PMC3924079

[jane12456-bib-0062] Ozgul, A. , Childs, D.Z. , Oli, M.K. , Armitage, K.B. , Blumstein, D.T. , Olson, L.E. *et al* (2010) Coupled dynamics of body mass and population growth in response to environmental change. Nature, 466, 482–485. 20651690 10.1038/nature09210 PMC5677226

[jane12456-bib-0063] Peel, A.J. , Pulliam, J. , Luis, A. , Plowright, R. , O'Shea, T. , Hayman, D. *et al* (2014) The effect of seasonal birth pulses on pathogen persistence in wild mammal populations. Proceedings of the Royal Society B: Biological Sciences, 281, 20132962. 24827436 10.1098/rspb.2013.2962 PMC4046395

[jane12456-bib-0064] Perelson, A.S. (2002) Modeling viral and immune system dynamics. Nature Reviews Immunology, 2, 28–36. 10.1038/nri700 11905835

[jane12456-bib-0065] Rees, M. , Childs, D.Z. & Ellner, S.P. (2014) Building integral projection models: a user's guide. Journal of Animal Ecology, 83, 528–545. 24219157 10.1111/1365-2656.12178 PMC4258094

[jane12456-bib-0066] Rees, M. , Childs, D.Z. , Rose, K.E. & Grubb, P.J. (2004) Evolution of size‐dependent flowering in a variable environment: partitioning the effects of fluctuating selection. Proceedings of the Royal Society of London B: Biological Sciences, 271, 471–475. 10.1098/rspb.2003.2596 PMC1691614 15129956

[jane12456-bib-0067] Saenz, R.A. , Quinlivan, M. , Elton, D. , MacRae, S. , Blunden, A.S. , Mumford, J.A. *et al* (2010) Dynamics of influenza virus infection and pathology. Journal of Virology, 84, 3974–3983. 20130053 10.1128/JVI.02078-09 PMC2849502

[jane12456-bib-0068] Vindenes, Y. & Langangen, Ø. (2015) Individual heterogeneity in life histories and eco‐evolutionary dynamics. Ecology Letters, 18, 417–432. 25807980 10.1111/ele.12421 PMC4524410

[jane12456-bib-0069] Waaijenborg, S. , Hahné, S.J. , Mollema, L. , Smits, G.P. , Berbers, G.A. , van der Klis, F.R. *et al* (2013) Waning of maternal antibodies against measles, mumps, rubella, and varicella in communities with contrasting vaccination coverage. Journal of Infectious Diseases, 208, 10–16. 23661802 10.1093/infdis/jit143 PMC4043230

[jane12456-bib-0070] Williams, J.L. , Miller, T.E. & Ellner, S.P. (2012) Avoiding unintentional eviction from integral projection models. Ecology, 93, 2008–2014. 23094372 10.1890/11-2147.1

