# Supplementary material for: Opportunities and challenges of Integral Projection Models for modelling host–parasite dynamics
Source: J Anim Ecol. 2015 Dec 1;85(2):343–55. doi: 10.1111/1365-2656.12456 (PMC4991293; doi:10.1111/1365-2656.12456)
Supplement: Supplementary file 1 — Figure S1. The time series of parasitaemia in the blood phase of malaria for five mice infected with 8 different clones, clone names shown as plot titles, and fitted linear regression predicted log infected RBCs as a function of current burden and time‐step either one time‐step ahead (dashed line) or over the full time course (solid line). Figure S2. The modelled density of log infected RBCs through time for each of the clones in the IPM. Earlier time‐steps are indicated by red colors, moving through to blue/purple for the final time‐steps – the density starts with a low mean log infected RBCs, which moves up, briefly, and then down. Table S1. Full set of parameters across all murine malaria clones (column headings) obtained from the regression models fitted to the time course of parasite density; see Table 1 for parameters common across clones. [file JANE-85-343-s001.docx]

**Figure S1:** The time series of parasitaemia in the blood phase of malaria for five mice infected with 8 different clones, clone names shown as plot titles, and fitted linear regression predicted log infected RBCs as a function of current burden and time-step either one time-step ahead (dashed line) or over the full time course (solid line).

**Figure S2:** The modelled density of log infected RBCs through time for each of the clones in the IPM. Earlier time-steps are indicated by red colors, moving through to blue/purple for the final time-steps – the density starts with a low mean log infected RBCs, which moves up, briefly, and then down.

**Table S1:** Full set of parameters across all murine malaria clones (column headings) obtained from the regression models fitted to the time course of parasite density; see Table 1 for parameters common across clones.

|  | **AD** | **AJ** | **AQ** | **AS** | **AT** | **BC** | **CW** | **ER** |
| --- | --- | --- | --- | --- | --- | --- | --- | --- |
| *a_g_* | 4.8324 | 5.7874 | 5.5148 | 4.5868 | 6.4701 | 4.8507 | 5.5967 | 4.7661 |
| *b_g_* | -0.1124 | -0.5291 | -0.1606 | -0.3229 | -0.2476 | -0.2940 | -0.2711 | -0.1037 |
| *c_g_* | 0.5200 | 0.5498 | 0.4600 | 0.6085 | 0.3820 | 0.5646 | 0.4847 | 0.5311 |
| *d_g_* | -0.0004 | 0.0240 | 0.0015 | 0.0117 | 0.0070 | 0.0100 | 0.0077 | -0.0003 |
| *σ_g_* | 0.43 | 0.60 | 0.49 | 0.66 | 0.56 | 0.82 | 0.58 | 0.47 |
| *a_0_* | 6.75 | 6.38 | 6.75 | 6.54 | 6.94 | 6.72 | 6.59 | 6.54 |
| *σ_0_* | 0.37 | 0.38 | 0.25 | 0.12 | 0.19 | 0.34 | 0.19 | 0.31 |
